# Supplementary material for: Seroepidemiology of pertussis in Huzhou: A population-based, cross-sectional study
Source: PLoS One. 2024 May 20;19(5):e0303508. doi: 10.1371/journal.pone.0303508 (PMC11104605; doi:10.1371/journal.pone.0303508)
Supplement: Attachment — Submitted filename: Response to Reviewers.docx [file pone.0303508.s002.docx]

**Response to Reviewers**

We thank the editor and reviewers for their constructive inputs to our paper. We have now addressed all the comments. Please find our point-to-point responses.

**Comments**

**Reviewer #1:**

1.It is not indicated which vaccine preparation was used in the vaccinated individuals and whether all vaccinated people were given the same preparation. In fact the response to PT could be different in relation to different vaccine preparation.

**Response:** Thank you for your thoughtful review and valuable feedback on our manuscript. We appreciate your concern regarding the lack of specific information on the vaccine preparation used in the vaccinated individuals. We acknowledge the importance of this aspect and recognize that variations in vaccine formulations may indeed influence the immune response.

Our study population received different pertussis vaccines. According to the vaccine immunization system database, the pertussis vaccines administered to our study participants include both the DTaP (Diphtheria, Tetanus, acellular Pertussis) vaccine and the pentavalent vaccine. In our revised manuscript, we have provided detailed information on the specific vaccine preparations used in our study (please see line 102-104 in Marked Copy). This clarification will enhance the transparency of our findings and contribute to a more comprehensive understanding of the observed outcomes. We will explicitly mention this in the revised manuscript and discuss the potential impact of different vaccine formulations on the PT response in the discussion section.

2.Another point to consider is the execution of the tests if they have been carried out by the same laboratory. Can you specify that?

**Response:** Thank you for bringing up an important consideration. We would like to clarify that all our experimental tests were conducted by the same laboratory, namely Hangzhou Edicon Medical Laboratory Co., Ltd. The samples were outsourced to Adicon Testing Company, a certified testing facility with appropriate accreditation. Additionally, the testing was performed using the pertussis toxin IgG antibody detection kit from Euroimmun, ensuring uniformity and quality in the testing procedures.

We believe this information addresses your concern about the execution of the tests, and we are confident in the consistency and reliability of the results obtained through this process. (please see line 106-110 in Marked Copy).

3.Check that all microbial names are italicized.

**Response:** Thank you for your careful review and valuable feedback. We appreciate your attention to detail. In response to your comment regarding the formatting of microbial names, we have made sure that all microbial names are properly italicized in the revised manuscript.

**Reviewer #2:**

1) It is not clear how many urban districts and counties there are in the city of Huzhou. Why was only one urban district and county selected? How representative are the estimates of the city of Huzhou?

**Response:** Thank you for your thoughtful review and for raising important questions regarding our study. We appreciate your time and effort in providing constructive feedback. Here is our response to your concerns:

Huzhou city comprises three counties and two districts. According to a multi-stage stratified sampling, one county(Deqing County) and one district(Wuxing District) were selected, and local residents were then chosen as the study subjects through random sampling methods. (please see line 85-90 in Marked Copy).

We employed a method to assess the pertussis infection rate that aligns with those commonly used in current studies, Similar situations were observed in other regions, with estimated pertussis infection rates as follows: 7290/100,000 in Chongqing^[1]^, 4572.81/100,000 in Ningbo^[2]^, 9395/100,000 in Guangdong^[3]^, 18080/100,000 in Jiangsu^[4]^, and 6600/100,000 in the Netherlands^[5]^ yielding similar estimated infection rates, indicating a high prevalence of pertussis in the population. However, caution is warranted in all extrapolations, and we also address the limitations in the discussion section of the paper.

2) After randomly selecting the urban district and county, how did the authors select the participants? Did they visit households? How were households sampled? How many individuals were enrolled from each household? The methods section needs to elaborate on the sampling procedure.

**Response:** Thank you for your insightful question regarding our sampling procedure. We appreciate your diligence in seeking clarity on this important aspect of our study. Here is an elaboration on the sampling procedure:

One county(Deqing County) and one district(Wuxing District) were selected randomly. After that, randomly select a street within the chosen county/district. Then the study subjects were randomly selected from the community health examination population within September 1st to October 1st 2023. (please see line 85-90 in Marked Copy)We randomly selected our study participants between September 1st to October 1st 2023. Therefore, our study did not utilize household sampling methods. However, I believe household sampling methods are also a valuable approach. The household sampling method has inspired me greatly, and I plan to incorporate it into future research endeavors to ensure a more representative sample.

3) Although the study had nine age bands, the number of samples is very small to arrive at any meaningful inference.

**Response:** Thank you for your thoughtful consideration of our study. We appreciate your feedback on the number of samples within the nine age bands. We would like to address your concern and provide additional context:

We acknowledge that the sample size within each age band may be limited, and we understand the importance of a sufficient sample size for robust statistical analyses. In our study, the small sample sizes in certain age bands were influenced by practical constraints, including the availability of participants within each age group during the study period.

We determined the required sample size using the formula for sample size calculation in cross-sectional studies. Based on a Zhejiang province serum Anti-PT-IgG positivity rate of 33.32%^[6]^, with α=0.05, 1-β=0.90, and d=0.1p, the minimum required sample size was calculated using the formula N＝u_a_^2^p(1-p)/d^2^. The calculated minimum sample size was 769 individuals. Accounting for a 10% loss to follow-up, a minimum of 854 individuals needed to be surveyed.

We employed a method to assess the pertussis infection rate that aligns with those commonly used in current studies, Similar situations were observed in other regions, with estimated pertussis infection rates as follows: 7290/100,000 in Chongqing^[1]^, 4572.81/100,000 in Ningbo^[2]^, 9395/100,000 in Guangdong^[3]^, 18080/100,000 in Jiangsu^[4]^, and 6600/100,000 in the Netherlands^[5]^ yielding similar estimated infection rates, indicating a high prevalence of pertussis in the population. Our study sample is comparable to those of similar studies, with similar numbers in each age group. The results obtained are also similar, all indicating a high prevalence of pertussis in the population. However, caution is warranted in all extrapolations, and we also address the limitations in the discussion section of the paper. However, larger studies with greater sample sizes are indeed needed to provide more robust estimates. We will consider conducting larger-scale studies in the future. Thank you for your valuable input.

4) One sample size for such a large city is not sufficient. Authors should have divided the city into different zones/regions and should have calculated the sample size at region/zonal level. This would have enabled authors to look for any spatial variations within the city.

**Response:** We appreciate your thoughtful consideration of our study, and we acknowledge the importance of a robust sample size, especially in the context of a large city.

We understand your suggestion regarding dividing the city into different zones or regions to calculate sample sizes at a more granular level for exploring potential spatial variations. Huzhou is a small city of eastern China with about 3 million population. Huzhou city comprises three counties and two districts, which are close to each other. The economic development levels of each district and county are similar. In our study a multistage stratified random sampling method was employed to select the participants. Firstly, one county(Deqing County) and one district(Wuxing District) were selected. Wuxing District is located to the north of Huzhou, while Deqing County is situated to the south of Huzhou. Choosing these two areas provides a good representation of the entire city. After selecting Wuxing District and Deqing County, we further randomly selected one street communities in each area for the random selection of study subjects. Therefore, although the sample size in this study is relatively small, it can effectively represent the entire city region.

In the revised manuscript, we will address this concern by discussing the limitations associated with the potential implications for spatial variations. Additionally, we will explore opportunities to collaborate with local authorities and institutions to conduct future studies with a more refined spatial sampling strategy.

5) How the number of participants per age group was fixed? Does it represent the age structure of the city population?

**Response:** Thank you for raising an important question regarding the number of participants per age group in our study. We appreciate your scrutiny, and we would like to provide clarification on this matter.

We determined the required sample size using the formula for sample size calculation in cross-sectional studies. Based on a Zhejiang province serum Anti-PT-IgG positivity rate of 33.32%^[6]^, with α=0.05, 1-β=0.90, and d=0.1p, the minimum required sample size was calculated using the formula N＝u_a_^2^p(1-p)/d^2^. The calculated minimum sample size was 769 individuals. Accounting for a 10% loss to follow-up, a minimum of 854 individuals needed to be surveyed.

We determined the age groups of the population by reviewing previous studies. Since children are the high-risk group for pertussis, bearing a heavier burden of the disease, we predominantly selected children aged below 10 years, especially those under 24 months. Children under 10 years old accounted for 50.54% of the total study population.

Based on the 2020 census data (The specific age distribution data from the seventh population census of Huzhou City can be found in the attached table), the age distribution of our study sample differs from that of the general population. We primarily focused on recruiting more children participants due to the higher incidence of pediatric cases. This discrepancy presents a limitation of our study, which we will address in the discussion section. We will emphasize that extrapolating our findings to the broader population may be limited, and caution is needed when interpreting the results.

6) I suggest the authors use the STROBE checklist for cross-sectional studies

**Response:** Thank you for your valuable suggestion. Following your advice, I have reviewed our manuscript using the STROBE cross-sectional checklist, made necessary modifications and improvements to the manuscript, and attached the completed STROBE checklist as supplementary material.

**Reviewer #3**

Thank you very much for being able to review our manuscript, and to give valuable suggestions on the revision, which is very helpful for us to improve the quality of the manuscript. We have carefully read each of your suggestions and revised them accordingly in the paper. The following is our reply.

1.Line 18: Consider rewording the sentence

**Response:** Thank you for your suggestion. We have revised the sentence in question (Line 18) in the manuscript for improved clarity and expression.

2.Line 34: Consider representing the infection rate as a whole number while still including the 95% CI to the hundredth place

**Response:** Thank you for your constructive suggestion. We have revised Line 34 and other parts of the manuscript in accordance with your advice. The infection rates in the manuscript were represented as a whole number, and the 95% confidence interval has been specified to the hundredth place.

3.Line 56: What age groups are used to signify infants, adolescents, and adults? Does Adults include +65 years of age?

**Response:** Thank you for your valuable suggestion. According to previous literature^[7]^, infants typically refer to those aged 1 year and below, adolescents refer to individuals between 10 and 19 years old, adults refer to those aged 20 and above, including individuals aged 65 and older.

4.Line 61: Please explain what you mean that pertussis toxin is a protective antigen. Yes, it is unique to bordetella pertussis thus specific but I don't understand what you mean it is protective antigen when it is an exotoxin that inhbiits neutrophil and macrophage recruitment in the early stages of infection.

**Response:** Thank you for bringing attention to Line 61. We appreciate your clarification request regarding the term "protective antigen" in the context of pertussis toxin.

The term "protective antigen" refers to an antigenic component that, upon invasion of the host by a pathogenic microorganism, induces a protective immune response by the host's immune system.

The term "protective antigen" when referring to pertussis toxin primarily stems from its role in pertussis vaccines. In the context of pertussis vaccination, pertussis toxin is utilized as an antigen to stimulate the production of specific antibodies in the host. These antibodies, elicited by the presence of pertussis toxin in the vaccine, enhance the immune system's ability to combat the pertussis bacterium.

The term emphasizes the vaccine's capacity to induce a protective immune response. Although pertussis toxin may exert some pathogenic effects during infection, its inclusion in the vaccine is intended to harness its antigenic properties to promote an effective immune defense. The antibodies generated in response to the vaccine can rapidly recognize and neutralize pertussis bacteria upon subsequent exposure, thus preventing or mitigating the progression of the disease.

In essence, the designation "protective antigen" underscores its role in inducing an immune response that confers protection against specific diseases when incorporated into a vaccine formulation.

5.Line 85: Consider different phrasing instead of 'so on'

**Response:** Thank you for your suggestion regarding Line 85. We appreciate your attention to detail and agree that the phrase "so on" could be replaced with more precise wording.

In response to your feedback, we have revised the manuscript to provide a more specific and clear description, avoiding the use of "so on." We have changed “so on” with other relevant factors.

6.Line 116: Maternal antibodies typically clear in the first 6 months, does the analysis change if you looked at greater than or equal to 2 years? Is the 3 year criteria based more on the 4th dose at 18 months?

**Response:** Thank you very much for your thorough review and valuable suggestions. We fully agree with your observation that maternal antibodies are typically cleared within the first 6 months after birth. Our choice to evaluate the infection rate of pertussis (whooping cough) in individuals aged 3 years and above aims to exclude the influence of vaccination. We have revised this sentence in our manuscript. In our country, the pertussis vaccination schedule includes doses administered at 3, 4, and 5 months after birth, followed by a booster dose at 18 months. Within the first year after vaccination, it is challenging to differentiate between antibodies formed due to natural infection and those induced by vaccination. Therefore, we opted to assess the infection rate in individuals aged 3 years and older to mitigate the impact of vaccination. Selecting individuals aged 2 years and above might introduce bias due to the effect of vaccination, potentially overestimating the infection rate, particularly in the younger age groups.

7.Line 141: With the schedule being 3,4,5 months is it known if any of the susceptible cases where unvaccinated or only received 1 dose of vaccine?

**Response:** Thank you for your inquiry. We have conducted a detailed analysis and found that among the susceptible individuals between months 4 to 23, there were a total of 18 cases. Among them, one had no vaccination history, eight received one dose, four received two doses, three received three doses, and two received four doses.

8.Table 1: Do the authors have any conclusions as to why the Age 30-39Y group has no acute infections while all other groups have acute infections.

**Response:** Thank you for your observation regarding Table 1. Upon careful consideration, we believe that the absence of acute infections in the Age 30-39Y group could be attributed to several factors, including but not limited to the relatively smaller sample size in this specific age group.

One possible explanation is that the lower number of individuals in the Age 30-39Y group may have influenced the statistical outcomes, resulting in the apparent absence of acute infections. Additionally, variations in exposure patterns, immunity levels, or other demographic factors within this age group could contribute to the observed trend.

To address this concern, we will thoroughly examine the data, explore potential influencing factors, and provide a more detailed discussion in the revised manuscript.

9.Table 1: There is a type in the Table 40-4Y instead of 40-49Y

**Response:** Thank you for your reminder. There was a typographical error, and it has been corrected in the manuscript.

10.Table 2: It is not clear if any of the 0-3 months (Total 97 enrolled) are included in this table. Would that group represent a large portion of the zero vaccines group?

**Response:** Thank you for your inquiry regarding Table 2. We appreciate your attention to detail. The 0-3 months group is indeed included in Table 2, and it primarily represents a significant portion of the zero vaccines group.

In the 0-3 month age group in our study, there were 50 individuals with no vaccination history, 22 individuals who received one dose, 5 individuals who received two doses, and vaccination history was unknown for 20 individuals.

In our revised manuscript, we will ensure clarity on the inclusion of the 0-3 months group in Table 2 and provide a more explicit description of its representation within the zero vaccines category.

11.Line 235: I don't think this is a correct assumption. There is no mention of matched infant-mother sera to establish low transfer between the mother and infant. Maternal antibody transfer is primarily based on the mother's antibody level for pertussis and there is active transport during the third trimester of pregnancy. Additionally protective suggests functional antibody which is not what the ELISA measures. You go on to explain the benefits of maternal immunization in other countries correctly but the initial statement of low transfer is not proven in this paper. Please refine the statement in Line 235

**Response:** Thank you for your careful attention. Indeed, our assumption at line 235 was inappropriate. We can only infer that the levels of pertussis toxin antibodies in infants who have not yet reached the age for vaccination are very low. Since our country does not have a strategy for vaccinating adults or pregnant women against pertussis, it is very likely that maternal levels of pertussis toxin antibodies are also low. As our study found, pertussis toxin antibody levels in women of childbearing age are generally low.

In fact, pertussis toxin antibodies are readily transferred across the placenta with high efficiency,^[8]^ resulting in infant serum antibody levels that are essentially equivalent to those of the mother. Additionally, the half-life of transplacental antibodies is relatively short, approximately 6 weeks. However, due to the lack of maternal booster vaccination against pertussis during pregnancy, maternal antibody levels during pregnancy are low, and transplacental antibody levels rapidly decline, leading to low levels of protective antibodies in the infant's body.^[9]^

Clinical trials of pertussis vaccines typically rely on measuring levels of pertussis toxin antibodies to reflect the immune response to vaccination. It is generally considered that a fourfold increase in pertussis toxin antibody levels post-vaccination indicates the presence of protective antibodies in the body. Pertussis toxin antibody levels can also serve as a good indicator of the body's protective antibody levels. The ELISA assay kit for detecting pertussis toxin antibodies is a widely used method internationally.

We appreciate your thorough review and constructive comments, which contribute significantly to the precision and clarity of our manuscript.

12.Line 276: Are you referring to correlates of protection or fuctionally protective antibodies?

**Response:** Thank you for your inquiry regarding Line 276. Your question is insightful. In this statement, we are referring to the absence of established standards for positive level of Anti-PT-IgG, making it challenging to evaluate the positive rate of Anti-PT-IgG of different population.

[1]	YAO N, ZENG Q, WANG Q. Seroepidemiology of diphtheria and pertussis in Chongqing, China: serology-based evidence of Bordetella pertussis infection [J]. Public Health, 2018, 156: 60-66.

[2]	ZHAO X F, YE S, MA R, et al. Seroepidemiology of pertussis in healthy population in Ningbo, 2019 [J]. Zhonghua Liu Xing Bing Xue Za Zhi, 2021, 42(4): 638-642.

[3]	ZHANG Q, ZHENG H, LIU M, et al. The seroepidemiology of immunoglobulin G antibodies against pertussis toxin in China: a cross sectional study [J]. BMC infectious diseases, 2012, 12(1): 1-7.

[4]	CHEN Q, WANG W, SHI X, et al. Seroepidemiology of pertussis in the east of China: Estimates of incidence of infection in adolescents and adults pre-and post-COVID-19 [J]. Frontiers in Public Health, 2022, 10: 1054617.

[5]	DE MELKER H E, VERSTEEGH F G, SCHELLEKENS J F, et al. The incidence of Bordetella pertussis infections estimated in the population from a combination of serological surveys [J]. J Infect, 2006, 53(2): 106-113.

[6]	HE H, YAO P, ZHOU Y, et al. Is Pertussis Infection Neglected in China? Evidence from a Seroepidemiology Survey in Zhejiang, an Eastern Province of China [J]. PLoS One, 2016, 11(5): e0155965.

[7]	NIEVES D J, HEININGER U. Bordetella pertussis [J]. Microbiol Spectr, 2016, 4(3).

[8]	VAN SAVAGE J, DECKER M D, EDWARDS K M, et al. Natural history of pertussis antibody in the infant and effect on vaccine response [J]. J Infect Dis, 1990, 161(3): 487-492.

[9]	HEALY C M, MUNOZ F M, RENCH M A, et al. Prevalence of pertussis antibodies in maternal delivery, cord, and infant serum [J]. J Infect Dis, 2004, 190(2): 335-340.
